# Diabetogenic effect of pravastatin is associated with insulin resistance and myotoxicity in hypercholesterolemic mice

**DOI:** 10.1186/s12967-019-2045-6

**Published:** 2019-08-27

**Authors:** Estela Lorza-Gil, Marta García-Arevalo, Bianca Cristine Favero, Maria Cristina C. Gomes-Marcondes, Helena C. F. Oliveira

**Affiliations:** Department of Structural and Functional Biology, Biology Institute, State University of Campinas, Cidade Universitária Zeferino Vaz, Rua Monteiro Lobato, 255, Campinas, SP CEP 13083-862 Brazil

**Keywords:** Statins, Insulin resistance, Muscle proteolysis, Myotoxicity

## Abstract

**Background:**

HMG-CoA reductase inhibitors (statins) are cholesterol-lowering drugs widely used to treat hypercholesterolemia and prevent cardiovascular disease. Statins are generally well tolerated, but adverse reactions may occur, particularly myopathy and new onset of diabetes. The exact mechanism of statin-induced myopathy and diabetes has not been fully elucidated. We have previously shown that treatment of hypercholesterolemic (LDLr^−/−^) mice with pravastatin for 2 months decreased pancreatic islet insulin secretion and increased oxidative stress and cell death, but no glucose intolerance was observed. The purpose of the current work was to study long-term pravastatin effects on glucose homeostasis, insulin sensitivity, muscle protein turnover and cell viability.

**Methods:**

LDLr^−/−^ mice were treated with pravastatin for 3, 6 and 10 months. Glucose tolerance, insulin resistance and glucose-stimulated insulin secretion were evaluated. The rates of protein synthesis and degradation were determined in gastrocnemius muscle after 10 months of treatment. Insulin signalling, oxidative stress and cell death were analysed in vitro using C2C12 myotubes.

**Results:**

After 6 and 10 months of treatment, these mice became glucose intolerant, and after 10 months, they exhibited marked insulin resistance. Reduced islet glucose-stimulated insulin secretion was observed after the 3rd month of treatment. Mice treated for 10 months showed significantly decreased body weight and increased muscle protein degradation. In addition, muscle chymotrypsin-like proteasomal activity and lysosomal cathepsin were markedly elevated. C2C12 myotubes exposed to increasing concentrations of pravastatin presented dose-dependent impairment of insulin-induced Akt phosphorylation, increased apoptotic markers (Bax protein and cleaved caspase-3) and augmented superoxide anion production.

**Conclusions:**

In addition to reduced insulin secretion, long-term pravastatin treatment induces insulin resistance and muscle wasting. These results suggest that the diabetogenic effect of statins is linked to the appearance of myotoxicity induced by oxidative stress, impaired insulin signalling, proteolysis and apoptosis.

## Background

Statin therapy is effective for lowering cholesterol and decreasing cardiovascular mortality [1]. These drugs are among the most prescribed drugs in Western countries; they are taken by more than 25 million individuals worldwide [2]. Statins competitively inhibit 3-hydroxy-3-methylglutaryl CoA (HMG-CoA) reductase, thus reducing endogenous cholesterol synthesis [3]. The beneficial effects of statins are associated not only with lipid-lowering capacity but also with other pleiotropic actions, such as improved endothelial function, reduced vascular inflammation, and antioxidant effects [4]. Although statins are generally well tolerated, in recent years, some dose- and class-dependent side effects have been reported. Emerging evidence suggests that long-term statin treatment is associated with type 2 diabetes mellitus occurrence, as indicated by large-scale meta-analyses [5, 6]. Statins could lead to diabetes by increasing insulin resistance, impairing beta cell function or a combination of these two processes [7]. Our group previously demonstrated, in a familial hypercholesterolemia model (LDLr^−/−^ mice), that chronic pravastatin treatment resulted in beta cell dysfunction associated with reduced insulin exocytosis and increased beta cell oxidative stress and death [8, 9]. Studies relating statin therapy and insulin sensitivity are controversial [10, 11]. A meta-analysis by Baker and colleagues showed that while pravastatin improves insulin sensitivity, atorvastatin, simvastatin and rosuvastatin worsen insulin sensitivity [12]. Experimental studies indicate that statins induce insulin resistance. In adipocytes, atorvastatin leads to the reduced expression of GLUT4 in vivo and in vitro [13], and simvastatin decreases IGF-1 signalling (pAKT, pERK) in muscle cells [14] and impairs the classical insulin signalling pathway and glucose uptake in myotubes [15, 16]. Simvastatin was shown to cause insulin resistance in mice and impaired glucose uptake in C2C12 myotubes by diminishing the activation of AKT by mTORC2 and downstream effects on GSK3, impairing the translocation of GLUT4 and causing atrophy of C2C12 myotubes [17, 18].

Muscle symptoms, such as fatigue, pain or weakness, are the most common statin side effects: these symptoms occur in up to 7% of statin users and up to 25% of statin users who participate in vigorous physical exercise [19]. Previous studies have shown that statin-induced muscle dysfunction is related to impaired mitochondrial function [20–22], protein breakdown [23], reduced protein synthesis [24], decreased lipid uptake and synthesis [25] and increased ectopic lipid deposition [26]. Skeletal muscle accounts for the major glucose disposal site in the body, and impaired muscle viability or glucose uptake may result in a risk of diabetes. Skeletal muscle is also the main protein reservoir in the body. Protein levels in skeletal muscle are determined by the insulin-mediated dual regulation of protein synthesis and protein degradation [27]. Impairment of insulin-stimulated phosphoinositol 3-kinase/Akt signalling is suggested to increase protein degradation in skeletal muscle [28] and may lead to loss of skeletal muscle mass and function [29].

Pravastatin is one of the less myotoxic statin classes [20, 30], but few experimental studies have followed the long-term effects of pravastatin. In the present study, we hypothesized that the pravastatin-induced risk of diabetes is connected to muscle insulin resistance and toxicity. In addition, most experimental studies use normolipidaemic models, which may not be the correct biological context to study HMG-CoA reductase inhibitors. Our previous studies [8, 9] showed that 2 months of pravastatin treatment of the hypercholesterolemic LDLr^−/−^ mice led to pancreatic islet toxicity, but no glucose intolerance was observed. Here, we show that after long-term pravastatin treatment, these mice develop glucose intolerance in association with insulin resistance, muscle protein degradation and cell oxidative stress and death.

## Methods

### Animals

Low-density lipoprotein receptor knockout (LDLr^−/−^) female mice with a C57BL/6J background, originally from the Jackson Laboratory (Bar Harbor, ME), were obtained from the breeding colony of State University of Campinas Multidisciplinary Center for Biological Research in Laboratory Animals (CEMIB/UNICAMP, Campinas, Brazil). Animal experiments were approved by the University’s Committee for Ethics in Animal Experimentation (CEUA/UNICAMP, protocol # 3819-1), and all experiments were performed in accordance with the national Brazilian guideline number 13 for “Control in Animal Experiments”, published on September 13th, 2013 (code 00012013092600005, available at 〈http://portal.in.gov.br/verificacao-autenticidade〉). The mice were kept under standard laboratory conditions (at 20–22 °C and a 12 h/12 h light/dark cycle) in the local (conventional) animal facility in individually ventilated cages (3–5 mice/cage), with free access to filtered tap water and regular rodent AIN93-M diet (standard laboratory rodent chow diet, Nuvital CR1, Colombo, PR, Brazil). Female mice (4 weeks old) were randomly assigned to six groups (3 treated and 3 control groups) according to the time of treatment with pravastatin (dissolved in the drinking water, 400 mg/L) for three (n = 6), six (n = 6) and ten (n = 5) months. Controls received filtered tap water without pravastatin. The pravastatin sodium (Medley, Sanofi, SP, Brazil) dose of 40 mg/kg body weight per day was based on drink consumption rates (3 mL/day). One out of 5 mice that belonged to the 10-month pravastatin treatment group died in the 7th month of treatment. Based on the current literature, the 40 mg/kg dose is considered a moderate effective dose of pravastatin for mice [31, 32], among protocols employing doses from 10 to 300 mg/kg body weight [33]. We previously characterized defective insulin secretion and beta cell death in this model treated with 40 mg/kg of pravastatin for 2 months [8, 9]. We chose to study female LDLr^−/−^ mice for several reasons, including increased susceptibility to atherosclerosis, increased plasma cholesterol levels and increased response to statin treatment in females compared with males. In addition, recent reports on the diabetogenic effects of statins demonstrate an increased risk for women compared with men. In terminal experiments, mice were anaesthetized via intraperitoneal injection of ketamine and xylazine (50 and 10 mg/kg) and euthanized by exsanguination through the retro-orbital plexus. All animal experiments were performed between 8:00 and 11:00 p.m.

### Plasma biochemical analyses

Blood glucose was measured using a glucose analyser (Accu-Chek Advantage; Roche Diagnostics, Basel, Switzerland). Plasma cholesterol and triglycerides were measured using standard commercial kits (Roche Diagnostics GMbH, Mannheim, Germany) according to the manufacturer’s instructions. Plasma insulin was measured using an Ultra-Sensitive Mouse Insulin ELISA Kit (Crystal Chemical, Illinois, USA). Fasting blood samples were obtained at 9:00 a.m. after a 12 h fasting period.

### Oral glucose tolerance test (OGTT) and insulin tolerance test (ITT)

Conscious and fasted mice (food removed at 8:00 p.m. and blood obtained between 8 and 9:00 a.m.) received an oral dose of glucose solution (1.5 g/kg body weight) by gavage. Blood samples were collected directly from the tail tip cut by the glucose analyser before (t = 0 min) and 15, 30, 60, and 90 min after the glucose load. One week after the GTT, the same mice were submitted to the ITT. Blood samples were collected from fasted mice that had been refed for 3 h (t = 0 min) and 5, 10, 15, 30 and 60 min after an intraperitoneal insulin injection (0.75 U/kg body weight, regular human insulin, Eli Lilly Co.) for glucose analysis.

### Pancreatic islet isolation and static insulin secretion

Pancreatic islets were isolated from overnight fasted and anaesthetized mice by collagenase type V (0.8 mg/mL; Sigma) digestion and were then selected under a microscope. Four replicates of 4 islets/well in each condition (basal and glucose stimulated) from each mouse were used for the insulin secretion assay. Islets were pre-incubated for 30 min at 37 °C in Krebs-bicarbonate buffer (KBB) composed of the following: 115 mmol/L NaCl, 5 mmol/L KCl, 2.56 mmol/L CaCl_2_, 1 mmol/L MgCl_2_, 10 mmol/L NaHCO_3_, and 15 mmol/L HEPES, supplemented with 5.6 mmol/L glucose and 0.3% BSA and equilibrated with a mixture of 95% O_2_/5% CO_2_ at pH 7.4. The islets were further incubated for 1 h in KBB containing glucose (2.8 or 11.1 mmol/L). At the end of the incubation period, insulin content was measured by radioimmunoassay [8, 9].

### Measurement of total protein synthesis and degradation

Protein synthesis rates were measured in gastrocnemius muscle excised from anaesthetized and exsanguinated mice using phenylalanine incorporation into proteins, and protein degradation rates were measured by tyrosine released into incubation medium, as previously described [34]. Briefly, right leg gastrocnemius muscle was used for protein synthesis, and left leg gastrocnemius was used for the protein degradation assay. For synthesis, muscle was pre-incubated for 30 min at 37 °C in Krebs–Henseleit bicarbonate (KHB) buffer (in mM: 110 NaCl, 25 NaHCO_3_, 3.4 KCl, 1 CaCl_2_, 1 MgSO_4_, 1 KH_2_PO_4_, 5.5 glucose and 0.01% albumin, pH 7.4) with continuous aeration (95% O_2_ and 5% CO_2_) and shaking. Next, KHB buffer was changed, supplemented with 5 µCi L[^3^H]-phenylalanine and incubated for 2 h. The muscles were then dried in filter paper, weighed, homogenized in trichloroacetic acid (TCA, 1:3 w/v), and centrifuged at 10,000 rpm for 15 min at 4 °C. The pellet was suspended in 1 M NaOH and incubated at 40 °C for 30 min. The supernatant was used to measure the total protein concentration and to quantity radioactivity in liquid scintillation using β-counter equipment (Beckman LS 6000 TA, Fullerton, CA, USA). Left gastrocnemius muscle was used for protein degradation analysis. Muscle was pre-incubated for 30 min at 37 °C in RPMI medium without phenol red under 95% O_2_–5% CO_2_ and shaking. Subsequently, muscles were incubated with KHB buffer containing cycloheximide, a protein synthesis inhibitor (130 µg/mL), for 2 h at 37 °C. Then, medium aliquots were treated with 30% TCA and centrifuged at 4000 rpm for 10 min to assess the tyrosine content using a fluorometric assay utilizing 20% 1-nitroso-2-naphthol reagent and nitric acid [34].

### Chymotrypsin-like and cathepsin enzyme activities

Chymotrypsin-like activity was determined in aliquots of homogenized muscle tissue by incubation with the fluorogenic substrate Suc LLVY-AMC (0.167 µg/mL in Tris–HCl, pH 7.4; excitation 360 nm, emission 460 nm). Cathepsin B activity was assayed using the fluorogenic substrate Z-Phe-Arg-NMec (0.02 mM with 0.1% Brij; excitation: 340–380 nm, emission: 460 nm) and cathepsin H, using Arg-NMec as substrate. Enzyme activities were expressed as units of fluorescence/µg protein/min [34].

### C2C12 cell culture

A murine myogenic cell line, C2C12, was obtained from the ATCC (ATCC.^®^. No. CRL-1772™; passage # 16–19). The cells were maintained in growth medium consisting of high glucose (4.5 g/L) Dulbecco’s modified Eagle’s medium (DMEM) supplemented with 10% foetal bovine serum (FBS) and 1% penicillin–streptomycin in a humidified cell culture incubator for 2 days or until 100% confluence. Afterwards, cell differentiation into myotubes was induced using high glucose DMEM supplemented with 2% horse serum for 5 days. After that, the cells were incubated with 1, 10, or 50 µM pravastatin sodium salt (Sigma) for 12 h. Then, the expression of proteins of interest was determined by Western blot analysis, and H_2_O_2_ and superoxide production rates were determined as described below. Since pravastatin is one of the least toxic statins due to its hydrophilic nature [21, 29, 35, 36], it was expected that we would need high doses of pravastatin to induce and observe both therapeutic and toxic effects. Sun et al. [36] showed that 1 and 10 µM pravastatin does not affect glucose metabolism in C2C12 cells. Thus, we used a wide dose range (1–50 µM) in the in vitro C2C12 studies.

### Western blot analysis

Cell homogenates were treated with Laemmli loading buffer containing dithiothreitol. After heating to 95 °C for 5 min, the proteins were separated by electrophoresis (30 µg protein/lane, 8 or 12% acrylamide/bisacrylamide gel) and were then transferred to nitrocellulose membranes. The nitrocellulose membranes were treated for 1.5 h with a blocking buffer (5% BSA, 10 mmol/L Tris, 150 mmol/L NaCl, and 0.02% Tween 20) and were incubated with the following primary antibodies: Caspase-3 (Millipore), Bax (Cell Signaling), Bcl-2 (Cell Signaling), phospho-ser^473^-AKT (Cell Signaling) and total AKT (Santa Cruz Biotechnology). GAPDH detected with rabbit polyclonal antibody and tubulin detected with mouse polyclonal antibody (Santa Cruz Biotechnology) were used as internal controls. Membranes were then incubated with horseradish peroxidase-conjugated secondary antibody (1:10,000, Invitrogen). Detection was performed using enhanced chemiluminescence (SuperSignal West Pico, Pierce, Rockford, IL). Band intensities were quantified by optical densitometry (Scion Image, Frederick, MD).

### Superoxide anion production

Myotube C2C12 cells (10^4^) were plated and differentiated in 96-well plates and treated with pravastatin 1, 10, or 50 µM in DMEM for 12 h. The cells were then washed and incubated in the dark for 30 min at 37 °C with 15 µM dihydroethidium (DHE) in DMEM. When oxidized by a superoxide anion, DHE produces 2-hydroxyethidium, which intercalates DNA, staining the nucleus with red fluorescence. After staining, the cells were washed with phosphate-buffered saline (PBS) followed by high glucose PBS (4.5 g/L). Fluorescence was monitored in a temperature-controlled (37 °C) SpectraMax M3 Microplate Reader (Molecular Devices) using the excitation and emission wavelengths of 535 and 635 nm, respectively. Images were taken by FLoid^®^ Cell Imaging Station (ThermoFisher).

### Hydrogen peroxide production

H_2_O_2_ release from cells was monitored by measuring the conversion of Amplex Red to the highly fluorescent resorufin in the presence of added horseradish peroxidase. A total of 10^4^ cells were incubated in 96-well plates, and after differentiation, the myotubes were treated with 1, 10 and 50 µM pravastatin for 12 h. Cells were incubated in a mixture containing 50 μM Amplex Red reagent (Invitrogen) and 0.1 U/mL horseradish peroxidase in Krebs–Ringer phosphate buffer (in mM: 145 NaCl, 5.7 sodium phosphate, 4.86 KCl, 0.54 CaCl_2_, 1.22 MgSO_4_, and 22 glucose, pH 7.35). This assay was conducted in the presence and absence of catalase (500 U/mL) for 1 h. Fluorescence was monitored over time in a temperature-controlled (37 °C) SpectraMax M3 Microplate Reader (Molecular Devices) using the excitation and emission wavelengths of 560 and 590 nm, respectively.

### Statistical analyses

Data are presented as the mean ± the standard error (SE), and the number (n) of mice or cell passage is indicated in each figure. Comparisons between two groups were analysed by unpaired Student’s t-test. Multiple comparisons were tested using one-way analysis of variance (ANOVA) with LSD (Fisher’s least significance difference) post hoc test (GraphPad Prism, RRID: SCR_002798; URL: http://www.graphpad.com). Animal and cell sample size (n) for each experiment was chosen based on similar studies in the literature. The level of significance was set at p ≤ 0.05.

## Results

LDLr^−/−^ mice, a model of human familial hypercholesterolemia, present high plasma cholesterol levels even under a low-fat diet, approximately 500 mg/dL (Table 1), due to an LDL receptor genetic deficiency and increased de novo cholesterol biosynthesis. We treated these mice with pravastatin, a hydrophilic statin, for 3, 6 and 10 months. Regardless of the time of treatment, the cholesterolaemia was significantly reduced by 30 to 45%. After 10 months of pravastatin treatment, the plasma triglyceride levels were also reduced by 40%. At this period of treatment, a significantly lower body weight was observed in treated mice (Table 1). Overnight fasting absolute values of glucose and insulin in plasma did not significantly vary between the control and pravastatin groups or with treatment time. However, taking into account the glucose/insulin ratio, the 10-month treatment group showed a 30% higher glycaemia per amount of insulin, which is suggestive of insulin resistance. This condition was further evaluated and confirmed by more appropriate tests, glucose and insulin tolerance tests, as described in the next section.

**Table 1:** Body weight and fasting plasma levels of lipids, glucose and insulin of LDLr^−/−^ mice treated with pravastatin for 3, 6 and 10 months

	3 months		6 months		10 months	
	Control	Pravastatin	Control	Pravastatin	Control	Pravastatin^a^
Body weight (g)	19.6 ± 0.4 (6)	19.17 ± 0.3 (6)	22.47 ± 0.2 (6)	22.05 ± 0.7 (6)	23.00 ± 0.3 (5)	21.55 ± 0.4 (4)*
Cholesterol (mg/dL)	584.6 ± 37.5 (6)	324.8 ± 28.6 (6)*	506.5 ± 22.8 (6)	272.0 ± 15.8 (6)*	514.7 ± 32.1 8 (5)	361.1 ± 46.7 (4)*
Triglycerides (mg/dL)	209.3 ± 44.7 (5)	148.4 ± 23.5 (5)	145.0 ± 17.1 (6)	124.9 ± 19.4 (6)	188.4 ± 22.2 (5)	108.1 ± 13.0 (4)*
Glucose (mg/dL)	89.20 ± 4.7 (6)	77.46 ± 3.4 (6)	96.3 ± 3.0 (6)	90.7 ± 4.4 (6)	92.4 ± 3.4 (5)	102.0 ± 6.6 (4)
Insulin (ng/mL)	0.44 ± 0.16 (4)	0.49 ± 0.10(4)	0.42 ± 0.03 (6)	0.45 ± 0.04 (6)	0.39 ± 0.06 (5)	0.33 ± 0.04 (4)
Glucose/insulin	188 ± 41 (4)	172 ± 38 (4)	267 ± 46 (6)	224 ± 27 (6)	258 ± 25 (5)	337 ± 49 (4)

Mean ± SE (n)

* p < 0.05 vs. control (untreated) mice, Student’s t-test

^a^One mouse died in the 7th month of treatment. Mice were fasted overnight

### Long-term pravastatin treatment impairs glucose and insulin homeostasis in LDLr^−/−^ mice

**Fig. 1:**
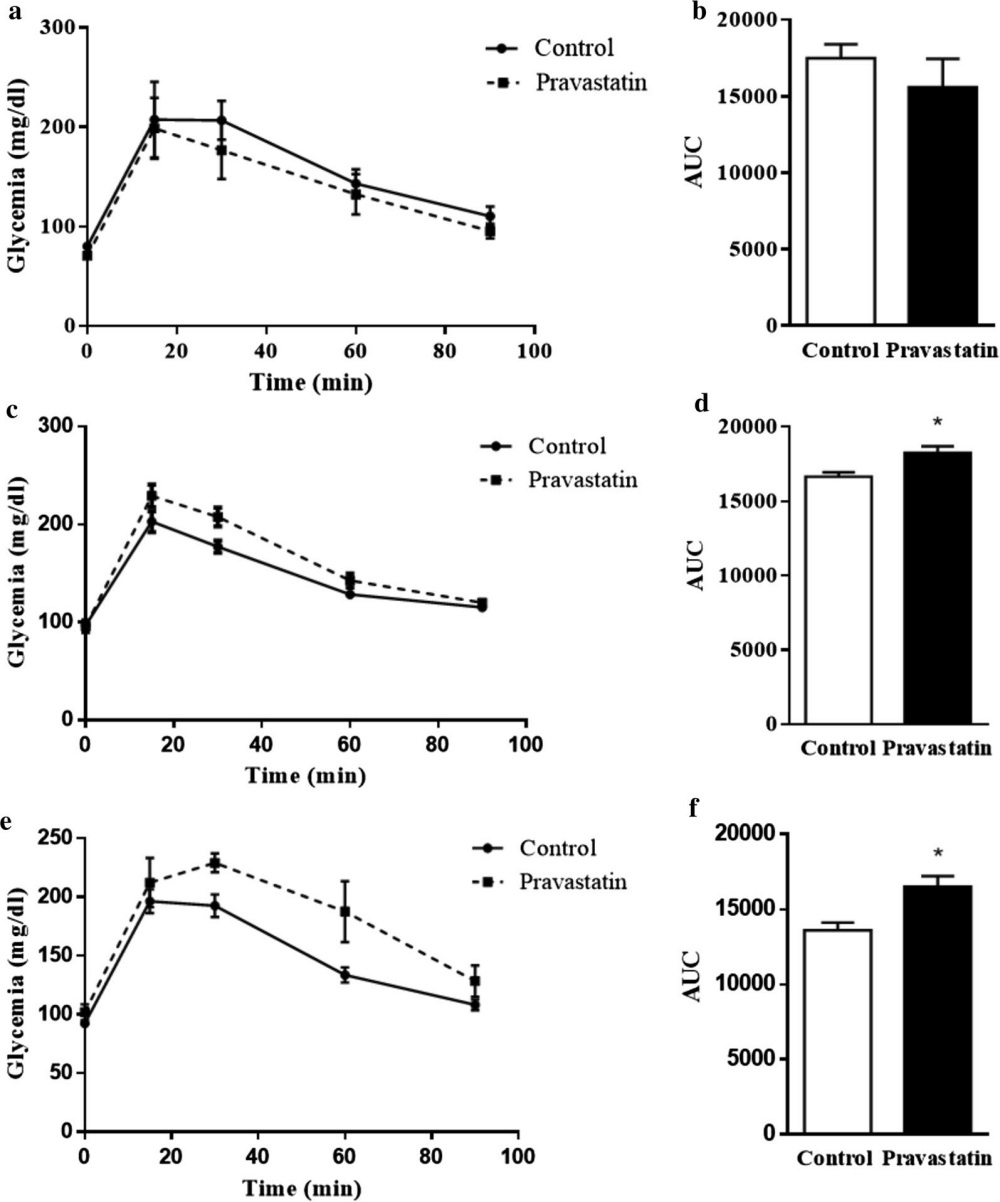
Long-term pravastatin treatment causes glucose intolerance in LDLr^−/−^ mice. A glucose tolerance test (GTT) was performed after 3 months (**a, b**), 6 months (**c, d**) and 10 months (**e, f**) of treatment. Blood glucose concentrations (**a, c** and **d**) and area under the curve (AUC) are represented (**b**, **d** and **f**). Mean ± SE. *p < 0.05 as determined by Student’s t-test between untreated and pravastatin-treated mice (n = 4–6)

**Fig. 2:**
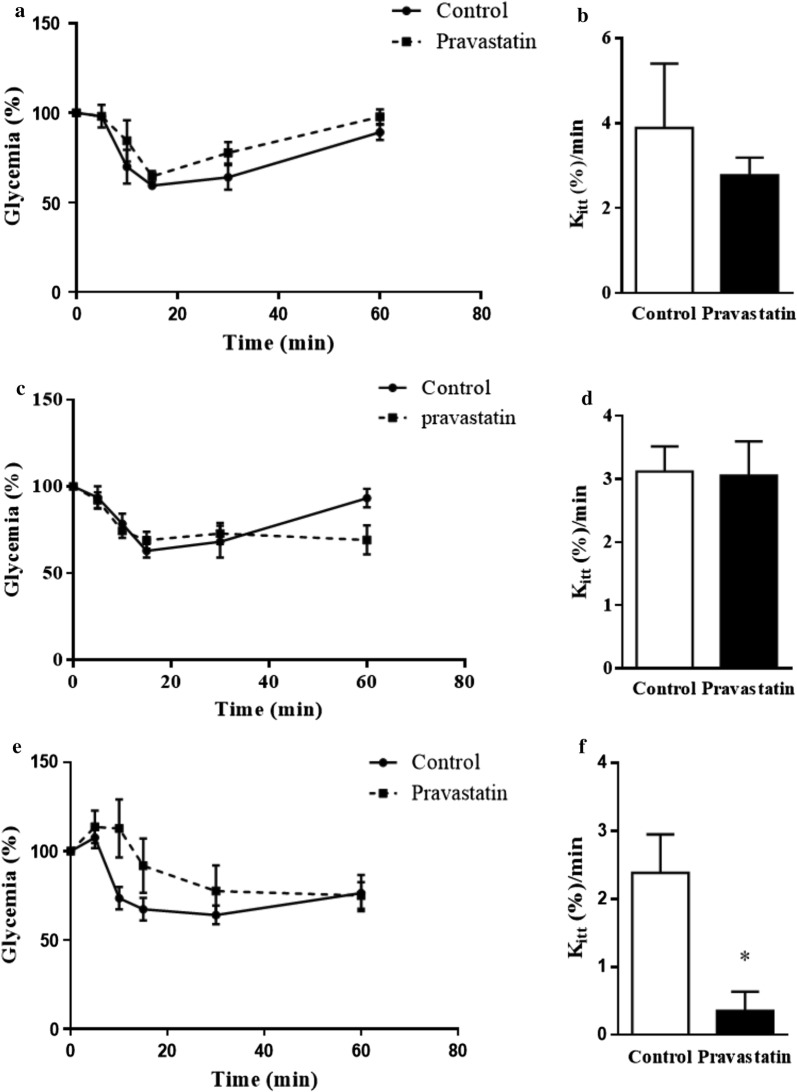
Long-term pravastatin treatment impairs insulin sensitivity in LDLr^−/−^ mice. Insulin tolerance tests (ITTs) were performed after 3 months (**a, b**), 6 months (**c, d**) and 10 months (**e, f**) of treatment. Blood glucose concentrations (%) (**a, c** and **e**), and disappearance rates (K_itt_)(**b**, **d**, and **f**) are shown. Mean ± SE. *p < 0.05 as determined by Student’s t-test between untreated (control) and pravastatin-treated mice (n = 4–6)

Glucose intolerance is a feature of type 2 diabetes. Therefore, we determined glucose tolerance (GTT) after 3, 6 and 10 months of pravastatin treatment. The mean areas under the glycaemia curves after an oral load of glucose were significantly increased in the groups of mice treated for 6 (35%) and 10 (55%) months with pravastatin compared with their respective non-treated control groups (Fig. 1). Thus, long-term pravastatin treatment induced glucose intolerance in LDLr^−/−^ mice. Global body insulin sensitivity was evaluated by the insulin tolerance test (ITT) performed in fasted mice that had been refed for 3 h. After insulin administration, the blood glucose disappearance rate during the first 10 min (K_itt_) markedly decreased (Fig. 2), and the area under the glycaemia curve significantly increased (~ 50%) in mice treated with pravastatin for 10 months (5496 ± 261 vs. 8150 ± 1086, n = 4–5, p < 0.05), characterizing an insulin resistance state induced by long-term treatment with pravastatin.

**Fig. 3:**
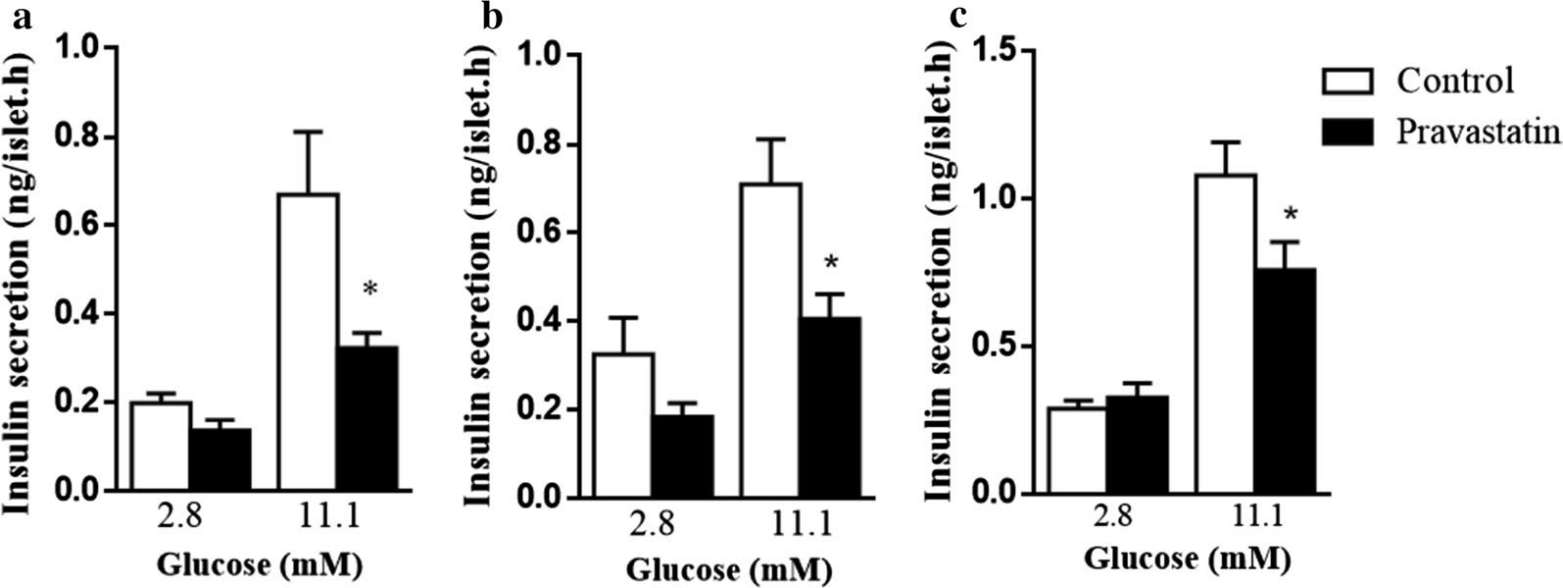
Long-term pravastatin treatment reduces ex vivo insulin secretion. Insulin secretion from isolated islets of LDLr^−/−^ mice in the presence of 2.8 or 11.1 mM glucose after 3 months (**a**), 6 months (**b**) and 10 months (**c**) of pravastatin treatment. Mean ± SE. *p < 0.05 as determined by Student’s t-test between untreated (control) and pravastatin-treated mice (n = 4–6 mice)

We have previously shown that ex vivo glucose-stimulated insulin secretion is impaired after 2 months of pravastatin treatment in LDLr^−/−^ mice [8]. Here, we observed that glucose (11.1 mM)-stimulated insulin secretion by isolated islets was decreased by 57%, 43% and 30% in mice treated with pravastatin for 3, 6 and 10 months, respectively, compared with non-treated mice (Fig. 3). Together, these results suggest that tissue insulin resistance needs to be established to induce glucose intolerance (6- and 10-month treatment), since pancreatic dysfunction without glucose intolerance was observed in the 3-month treatment.

### Long-term pravastatin treatment increases LDLr^−/−^ mouse muscle protein degradation

**Fig. 4:**
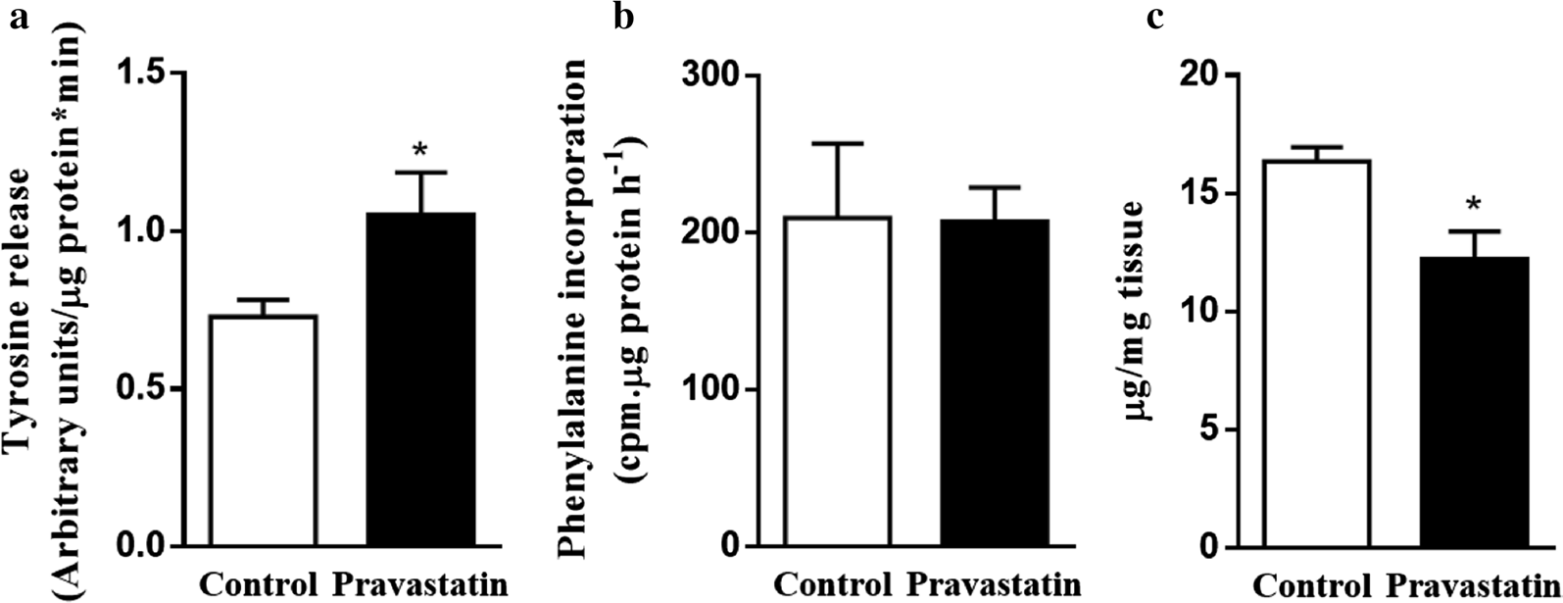
Long-term pravastatin treatment increases protein degradation in the gastrocnemius muscle of LDLr^−/−^ mice. **a** Rate of protein degradation in the gastrocnemius muscle of LDLr^−/−^ mice treated with pravastatin for 10 months, measured by the tyrosine released into the incubation medium. **b** Rate of protein synthesis in the gastrocnemius muscle of LDLr^−/−^ mice, measured by the ^3^H-phenylalanine incorporated into muscle proteins. **c** Total muscle protein concentrations in the gastrocnemius. Mean ± SE. *p < 0.05 as determined by Student’s t-test between untreated (control) and pravastatin-treated mice (n = 4–5)

To evaluate myotoxicity, we measured indicators of muscle protein turnover, such as protein synthesis and degradation rates. We observed that LDLr^−/−^ mice treated with pravastatin for 10 months presented disrupted muscle protein turnover due to increased gastrocnemius protein degradation as detected by greater tyrosine release (Fig. 4a) and no changes in protein synthesis rates (Fig. 4b). Increased protein degradation in the muscles of LDLr^−/−^ mice in the 10-month pravastatin treatment group was confirmed by markedly elevated chymotrypsin-like proteasomal activity (923 ± 89 vs 3470 ± 508 units/µg protein/h, n = 4, p = 0.008) and lysosomal cathepsin H (1.1 ± 0.2 vs 2.1 ± 0.2 units/µg protein/min, n = 4, p = 0.02). Accordingly, this long period of pravastatin treatment induced a decrease in the total gastrocnemius muscle protein content (Fig. 4c). The latter finding might explain the lower body weight found during this period (Table 1).

### Pravastatin impairs insulin signalling in C2C12 myotubes

**Fig. 5:**
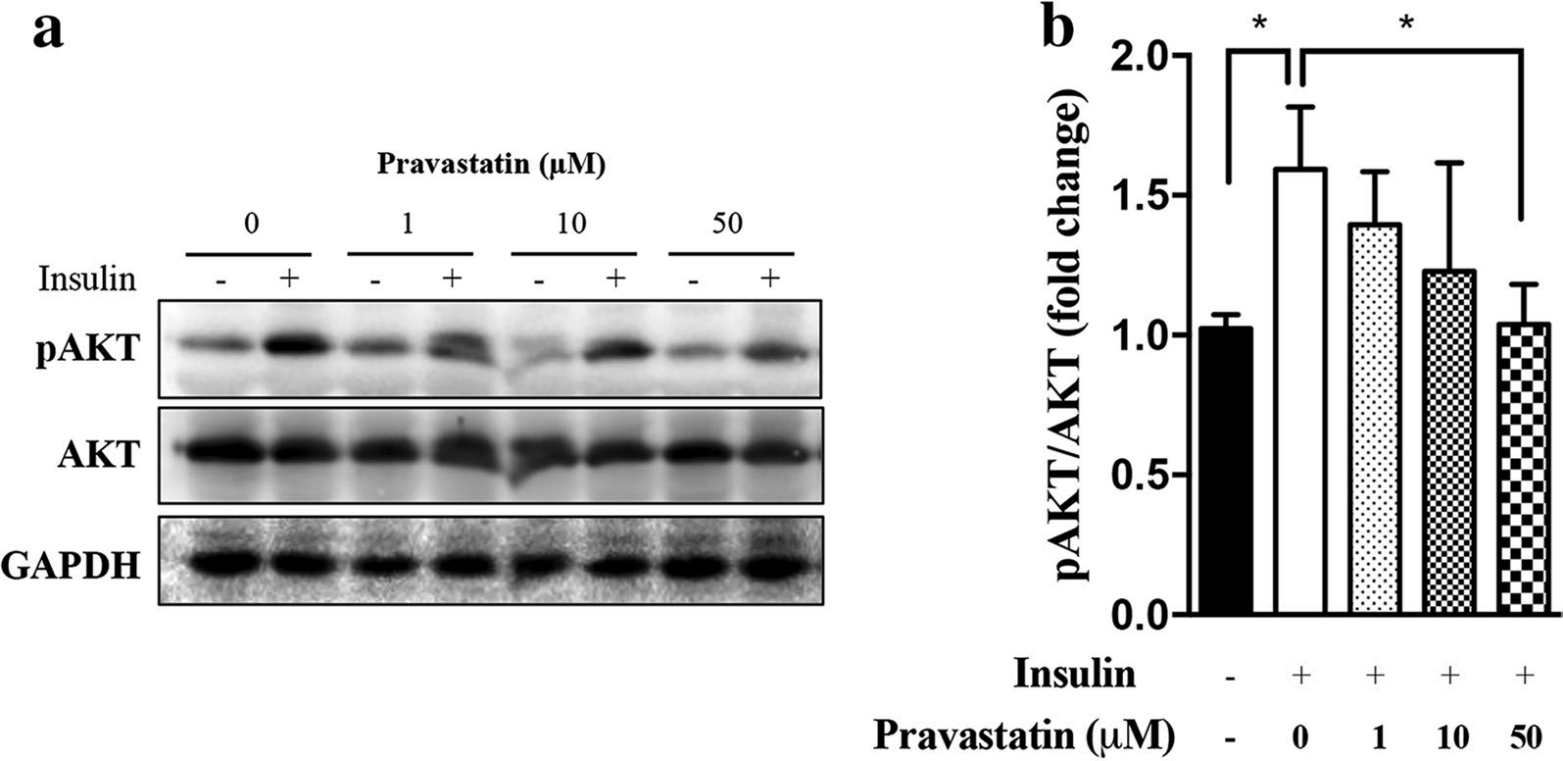
Pravastatin decreases insulin-induced AKT phosphorylation in C2C12 myotubes. **a** Representative Western blot of pAkt (Ser473), total Akt, and GAPDH in C2C12 myotubes treated with pravastatin (1, 10 or 50 µM) for 12 h. After pravastatin treatment, the myotubes were treated with 100 nM insulin for 10 min or left untreated. **b** The bar graphs depict the ratios of the intensities of the pAkt (Ser473) to Akt bands relative to the controls without insulin. Mean ± SE (three independent experiments). *p < 0.05 as determined by one-way analysis of variance (ANOVA) with LSD (Fisher´s least significance difference) post hoc test

Next, we investigated possible mechanisms involved in pravastatin-induced muscle protein degradation using a cell culture model, C2C12 myotubes. The differentiated C2C12 mouse cell line (myotubes) is a well-established in vitro model of skeletal muscle [37]. Impaired insulin activation of AKT reflects muscle insulin resistance and induces atrophy and apoptosis [17]. To test this possibility, C2C12 myotubes were pretreated with increasing concentrations of pravastatin (1, 10 or 50 µM) for 12 h and then exposed to insulin (100 nM) for 10 min. The effect of insulin on the phosphorylation of the serine 473 residue of AKT was significantly decreased in a pravastatin dose-dependent manner. Total AKT levels were not affected (Fig. 5a, b). These results indicate that pravastatin decreases insulin sensitivity in myotubes, confirming the observation in whole organisms (ITT, Fig. 2e, f) and previous studies with other statins. These findings may explain the loss of muscle mass in LDLr^−/−^ mice treated for a long period of time (Fig. 4c).

### Pravastatin induces oxidative stress and apoptosis in C2C12 myotubes

**Fig. 6:**
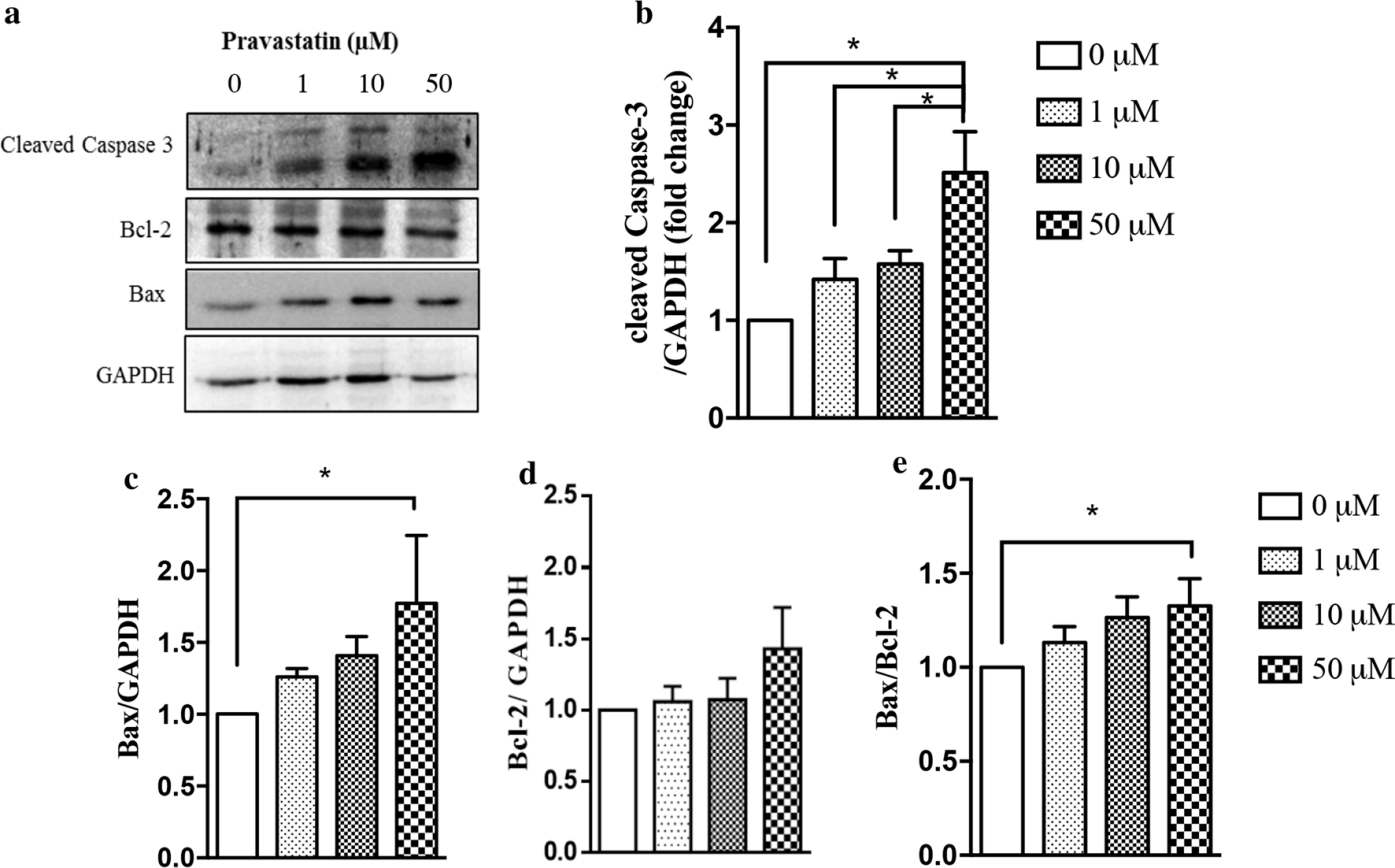
Pravastatin induces apoptosis in C2C12 myotubes. **a** Western blot analysis of cleaved caspase-3, Bcl-2 and Bax in C2C12 myotubes lysate with GAPDH as an internal control. **b**–**e** quantification of Western blot bands. Mean ± SE (three independent experiments). *p < 0.05 as determined by one-way analysis of variance (ANOVA) with LSD (Fisher´s least significance difference) post hoc test

To evaluate whether pravastatin induced muscle dysfunction, including cell death, we determined key markers of apoptosis in pravastatin-treated C2C12 myotubes. Cells treated with 50 µM pravastatin for 12 h showed an increased level of cleaved (activated) caspase-3 protein (Fig. 6a, b), the final apoptosis effector. In addition, the pro-apoptotic protein Bax was upregulated by pravastatin treatment (Fig. 6a, c), and the Bax/Bcl-2 ratio, used as an indicator of apoptotic potential, was increased in cells treated with 50 µM pravastatin (Fig. 6e).

**Fig. 7:**
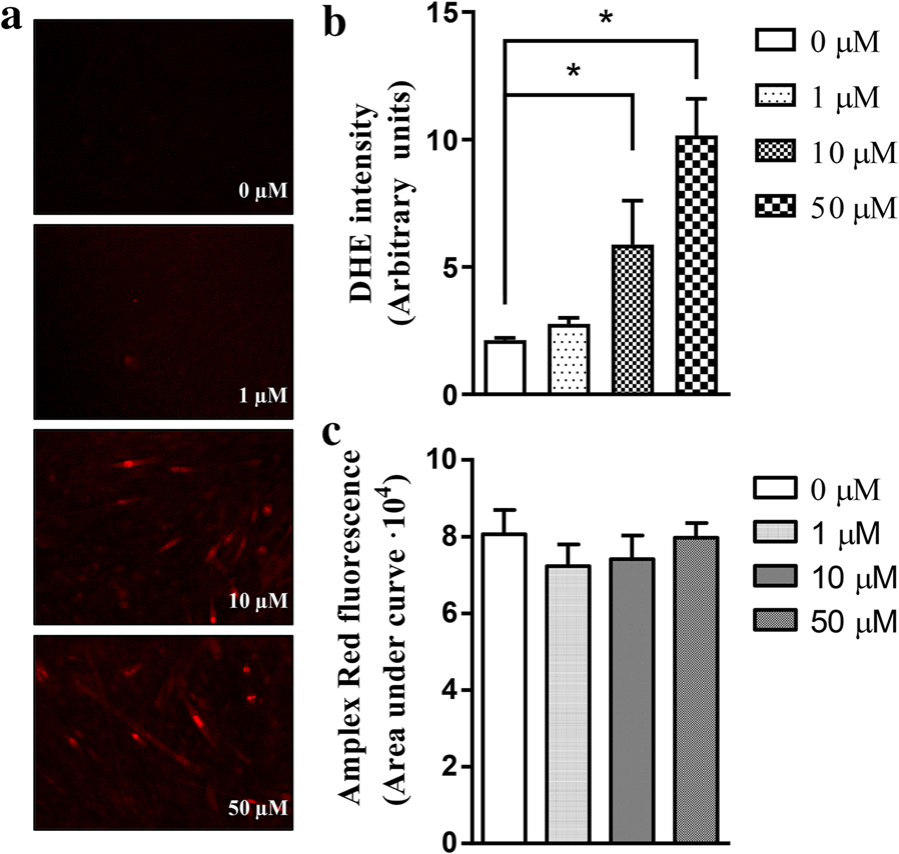
Pravastatin increases superoxide production in C2C12 myotubes. **a** Representative images showing in situ superoxide production as detected by the oxidation of dihydroethidium to fluorescent products (DHE; red fluorescence). **b** Quantification of DHE fluorescent products. **c** Quantification of the H_2_O_2_ released from cells using the Amplex Red probe during a 1-h incubation (areas under the curves). Mean ± SE, two independent experiments with six replicates in each experiment. *p < 0.05 as determined by one-way analysis of variance (ANOVA) with LSD (Fisher’s least significance difference) post hoc test

Several studies have shown that statins might impair mitochondrial function in skeletal muscle cells [20, 22, 38], and mitochondrial dysfunction is associated with both apoptosis and oxidative stress. Superoxide anions may be generated at several cell sites, including mitochondrial complex I and III of the electron transport chain. Thus, we measured global cellular superoxide using dihydroethidium (DHE), a probe attacked by superoxide that generates the fluorescent product hydroxyethidium. We observed an increase in the hydroxyethidium signal when C2C12 cells were treated with pravastatin for 12 h in a dose-dependent manner (Fig. 7a, b). Superoxide may be converted into other reactive radicals (hydroxyl or peroxynitrite) or H_2_O_2_ by cytosolic and mitochondrial superoxide dismutases. Thus, we also measured H_2_O_2_ cell release using the specific Amplex red cell impermeable probe. No differences in the release of H_2_O_2_ from pravastatin-treated cells were observed (Fig. 7c).

## Discussion

Previous studies from our laboratory showed that LDLr^−/−^ mice treated with pravastatin for 2 months presented an impaired glucose-stimulated insulin secretion capacity and islet cell death associated with oxidative stress [8, 9]. However, these pravastatin-treated hypercholesterolemic mice were not glucose intolerant. Here, we show that longer periods of pravastatin treatment (6 and 10 months) disrupted mouse glycaemic homeostasis due to the establishment of an additional defect in tissue insulin sensitivity. Thus, the impairment of insulin secretion together with insulin resistance induced the diabetes feature of glucose intolerance in these pravastatin-treated LDLr^−/−^ mice. We hypothesized that insulin resistance could be the result of the reported statin myotoxicity observed in some patients. Therefore, we further studied muscle protein mass and turnover in vivo. Indeed, augmented gastrocnemius protein degradation and lower protein content were verified in mice in the 10-month treatment group. In addition, C2C12 myotubes treated with pravastatin showed increased superoxide production, diminished insulin signalling (AKT phosphorylation) and increased apoptosis (upregulation of Bax expression and caspase-3 activation). The decreased insulin sensitivity of myotubes is in line with the in vivo observation of insulin resistance and with previous studies on other statins.

Recently, numerous clinical trials and meta-analyses have associated statin therapy with a risk of diabetes [39–43]. More specifically, data reveal a 10–12% increased risk of new-onset diabetes mellitus, particularly in intensive treatment regimens [7]. From all statins, pravastatin is usually described in the literature as less diabetogenic compared with other lipophilic statins [12, 44]. Nevertheless, other studies related pravastatin with unfavourable glycaemic parameters [8, 9, 45], muscle and liver toxicity [46, 47].

The most common side effects of statins are myopathies, ranging from mild weakness, myalgia and myositis to severe rhabdomyolysis [48–50]. Similar to the present results, studies in a rhabdomyosarcoma cell line showed that statins significantly reduce cell viability and markedly enhance the activity of caspase-3 in a concentration-dependent manner [30]. Hydrophilic statins, rosuvastatin and pravastatin, were less cytotoxic than hydrophobic statins (cerivastatin, simvastatin acid, fluvastatin, atorvastatin, lovastatin acid and pitavastatin) [30]. Most studies report that statin-induced muscle apoptosis is executed via the mitochondrial pathway, involving an increase in the Bax/Bcl-2 ratio, followed by the release of cytochrome c and activation of caspase-9 and caspase-3 [51]. For example, treatment of C2C12 cells with simvastatin or atorvastatin (10 or 50 μM) for 24 h increased the content of the cleaved forms of caspase-9 and caspase-3 and DNA fragmentation and induced an atrophic cell phenotype [17]. Primary human skeletal muscle cells treated with 5 μM simvastatin for 48 h showed an increased protein level of Bax, mitochondrial permeability transition and apoptosis [21]. Apoptosis signalling is essential and precedes protein degradation in the wasting of skeletal muscle during many catabolic conditions [52].

Several studies provide evidence that statin-induced myopathy is due to mitochondrial oxidative stress. Deltoid biopsies from patients with statin-induced myopathy exhibited high oxidant production and decreased mitochondrial biogenesis [53]. In primary human skeletal myotubes [21] and rat gastrocnemius biopsies [22], simvastatin induced mitochondrial oxidative stress, as shown by increased O_2_^·−^ production and/or H_2_O_2_ release. In the present study, pravastatin treatment induced an increase in O_2_^·−^ production in C2C12 myotubes, which likely contributed to apoptosis and myopathy. Recently, we demonstrated oxidant production and oxidative damage in the pancreatic islet [9], plantar muscle [43] and liver [44] of LDLr^−/−^ mice treated with pravastatin for 2 and 3 months.

We show here that both the ubiquitin–proteasome pathway (UPP) and lysosomal pathway are involved in pravastatin-induced muscle protein degradation. Other studies also suggest that the activation of the UPP contributes to statin-related myopathy. The UPP is tightly regulated and responsible for the recognition and degradation of the majority of proteins in skeletal muscle [54]. Urso and colleagues showed enhanced protein degradation via the ubiquitin–proteasome pathway in human skeletal muscle in response to atorvastatin treatment combined with exercise [55]. Atrogin-1, an ubiquitin protein ligase, was found to be increased in human skeletal muscle biopsies of simvastatin- or atorvastatin-treated patients with myopathy [23]. The authors also showed that C2C12 myotubes treated with lovastatin had increased atrogin-1 contents and muscle proteolysis rates [23].

The AKT signalling pathway is involved in cell proliferation and survival [56], mitochondrial integrity [57] and protein synthesis and degradation balance [58]. Wang and colleagues suggested a cause-effect relationship between insulin resistance and muscle protein degradation in diabetic db/db mice [28]. The authors showed that improving insulin signalling (PI3Kinase/pAkt) using rosiglitazone suppressed muscle proteolysis by decreasing the activities of caspase-3 and the ubiquitin–proteasome system [28]. Here, we showed that pravastatin treatment diminishes AKT phosphorylation in C2C12 myotubes and decreases whole body insulin sensitivity (ITT). These results do not seem to be specific for pravastatin since these findings were also observed in cells treated with other statins [15, 17, 59]. Decreased AKT phosphorylation is induced by increasing oxidant production; the exposure of C2C12 cultured myotubes to H_2_O_2_ decreases insulin-induced AKT phosphorylation and glucose uptake [60]. Akt activation blocks protein degradation by inhibiting forkhead box O (FoxO) transcription and the subsequent expression of muscle atrophy inducers such as atrogin-1 [61]. Bonifacio and colleagues showed that simvastatin treatment impaired AKT phosphorylation, reduced FoxO3a phosphorylation and consequently increased atrogin-1 mRNA in C2C12 myotubes and mouse skeletal muscle. These events were associated with skeletal muscle protein degradation and reduced protein content [17]. Decreased AKT phosphorylation, upregulated PTEN (phosphatase and tensin homologue on chromosome 10) and increased oxidative stress were also observed after prolonged treatment of wild type mice with rosuvastatin [62]. Decreases in phosphorylated AKT, mitochondrial dysfunction (increased lactate/pyruvate ratio and reduced ATP levels) and muscle wasting via protein degradation and mitochondrial autophagy were reported in atorvastatin-treated rats [63]. Our group recently reported the mitochondrial dysfunction and oxidative stress occur in the plantar muscle and liver of pravastatin-treated LDLr^−/−^ mice [43, 44]. Therefore, we have demonstrated that, although less toxic than hydrophobic statins, long-term treatment of hypercholesterolemia with pravastatin may also cause myopathy and diabetes.

This study improves the understanding of the mechanisms involved in the diabetogenic side effects of statins and uncovers possible intervention targets. Considering the major benefits of statins in reducing cardiovascular events, we propose that the new onset of diabetes induced by statins might be prevented by the co-administration of coenzyme Q10, a safe antioxidant, as we have shown previously in shorter (3 months) treatments [9, 43, 44], or by co-treatment with an insulin sensitizer.

## Conclusions

**Fig. 8:**
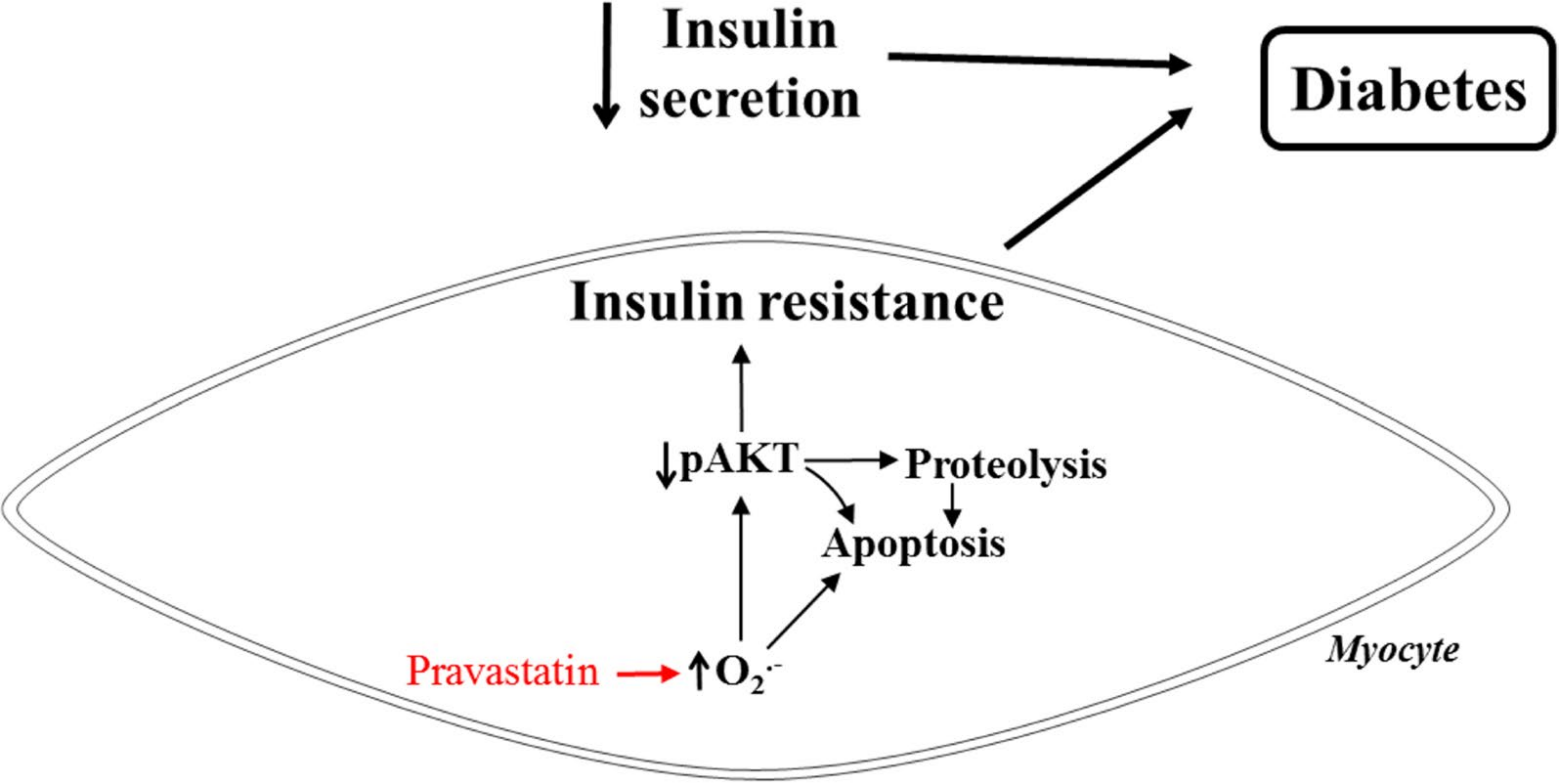
Proposed model of the diabetogenic effects of pravastatin. Oxidative stress induced by long-term treatment with pravastatin causes a reduction in the phosphorylation of Akt, which is permissive for proteolysis and apoptosis. Alternatively, oxidative stress may directly activate the apoptosis cell programme

In conclusion, we have demonstrated that, in addition to reduced pancreatic insulin secretion, long-term pravastatin treatment induces insulin resistance and muscle wasting. We thus propose that the diabetogenic effect of statins is linked to the appearance of myotoxicity induced by oxidative stress, impaired insulin signalling, proteolysis and apoptosis, as illustrated in Fig. 8.

## Data Availability

The datasets used and analysed in the current study are available from the corresponding author upon reasonable request.

## Abbreviations

AKT: serine/threonine-specific protein kinase, also known as protein kinase B
BSA: bovine serum albumin
DHE: dihydroethidium
DMEM: Dulbecco’s modified Eagle’s medium
ERK: extracellular signal-regulated kinases
FBS: foetal bovine serum
GAPDH: glyceraldehyde 3-phosphate dehydrogenase
GLUT: glucose transporter
HMG-CoA: 3-hydroxy-3-methylglutaryl coenzyme A
IGF-1: insulin-like growth factor-1
ITT: insulin tolerance test
KBB: Krebs-bicarbonate buffer
KHB: Krebs–Henseleit bicarbonate
LDL: low-density lipoprotein
LDLr: low-density lipoprotein receptor
mTORC2: mammalian target of rapamycin complex 2
OGTT: oral glucose tolerance test
PBS: phosphate-buffered saline
TCA: trichloroacetic acid
UPP: ubiquitin–proteasome pathway
